# Optimal Timing for Stereotactic Minimally Invasive Surgery in Supratentorial Spontaneous Intracerebral Hemorrhage With Tentorial Herniation: A Retrospective Study

**DOI:** 10.31083/RN38627

**Published:** 2025-11-30

**Authors:** Peijun Wu, Siying Ren, Guofeng Wu, Likun Wang

**Affiliations:** ^1^Emergency Department, The Affiliated Hospital of Guizhou Medical University, 550004 Guiyang, Guizhou, China

**Keywords:** minimally invasive stereotactic surgery, supratentorial cerebral hemorrhage, brain herniation, different time windows, cirugía estereotáctica mínimamente invasiva, hemorragia cerebral supratentorial, hernia cerebral, diferentes intervalos temporales

## Abstract

**Objective:**

To investigate the optimal timing of stereotactic minimally invasive surgery (SMIS) in individuals with supratentorial intracerebral hemorrhage (sICH) and brain herniation.

**Method:**

A retrospective analysis was conducted on patients with sICH and brain herniation who underwent SMIS in the emergency department of the Affiliated Hospital of Guizhou Medical University between January 2019 and October 2024. The patients were categorized into three groups based on the time from the onset of brain herniation to receiving SMIS: ≤6-h group (112 cases), 6–12-h group (57 cases), and >12-h group (32 cases). All enrolled patients were monitored over a 6-month period, and their prognoses were assessed using the Glasgow Outcome Scale Extended (GOSE), which was used for grouping. Clinical data, imaging findings, complications, comorbidities, infection markers, and outcome data were collected and analyzed comprehensively. Detailed analyses and comparisons were performed based on GOSE scores, Modified Rankin Scale (mRS) scores, and survival rates at 1, 3, and 6 months after sICH. Patients with mRS scores of 1–3 and GOSE scores of 4–8 had favorable outcomes. A detailed analysis of the six-month survival rate and post-treatment functional outcomes was conducted to draw research conclusions.

**Result:**

This study included 201 patients. At 6 months sICH, the mRS scores were 3.71 ± 1.30 for the ≤6-h group, 4.61 ± 1.25 for the 6–12-h group, and 4.18 ± 1.35 for the >12-h group, with the ≤6-h group showing markedly higher scores (*p* < 0.001). The GOSE scores at 6 months postoperatively were 4.05 ± 1.73 for the ≤6-h group, 3.05 ± 1.76 for the 6–12-h group, and 3.19 ± 1.73 for the >12-h group, with the ≤6-h group showed markedly higher scores (*p* = 0.001). The proportion of favorable outcomes at 6 months postoperatively was 47.3% for the ≤6-h group, 24.6% for the 6–12-h group, and 18.8% for the >12-h group, with the proportion of favorable outcomes highest in the ≤6-h group (*p* = 0.001). The Kaplan–Meier survival curve showed that the survival rate of the ≤6-h group was 80.4%, which was significantly higher than the 57.9% of the 6–12-h group and the 65.6% of the >12-h group (F = 10.060, *p* = 0.007).

**Conclusion:**

Undergoing SMIS intracranial hematoma evacuation within 6 h of brain herniation onset can effectively reduce neurological damage, significantly improve survival rates, and provide favorable prognosis.

## Abstract (es)

**Objetivo:**

Investigar el momento óptimo para la cirugía estereotáctica mínimamente invasiva (SMIS, stereotactic minimally invasive surgery) en personas con hemorragia intracerebral supratentorial (sICH, supratentorial intracerebral hemorrhage) y hernia cerebral.

**Método:**

Se realizó un análisis retrospectivo de pacientes con sICH y hernia cerebral que se sometieron a SMIS en el servicio de urgencias del Hospital Afiliado de la Universidad Médica de Guizhou entre enero de 2019 y octubre de 2024. Los pacientes se clasificaron en tres grupos de acuerdo con el tiempo transcurrido desde el inicio de la hernia cerebral hasta la SMIS: grupo ≤6 h (112 casos), grupo 6–12 h (57 casos) y grupo >12 h (32 casos). Todos los pacientes incluidos fueron monitorizados durante un periodo de 6 meses y sus pronósticos se evaluaron utilizando la Escala de Resultados de Glasgow Ampliada (GOSE, Glasgow Outcome Scale Extended), que se utilizó para la agrupación. Se recopilaron y analizaron de forma exhaustiva los datos clínicos, los resultados de las pruebas de diagnóstico por la imagen, las complicaciones, las comorbilidades, los marcadores de infección y los datos de los resultados. Se realizaron análisis y comparaciones detallados de acuerdo con las puntuaciones de la GOSE, las puntuaciones de la Escala de Rankin Modificada (mRS, Modified Rankin Scale) y las tasas de supervivencia a 1, 3 y 6 meses después de la sICH. Los pacientes con puntuaciones en la mRS de 1–3 y puntuaciones en la GOSE de 4–8 tuvieron resultados favorables. Se realizó un análisis detallado de la tasa de supervivencia a los seis meses y de los resultados funcionales tras el tratamiento a fin de extraer conclusiones de la investigación.

**Resultado:**

En este estudio se incluyeron 201 pacientes. A los 6 meses de la sICH, las puntuaciones mRS fueron de 3,71 ± 1,30 para el grupo ≤6 h, 4,61 ± 1,25 para el grupo 6–12 h y 4,18 ± 1,35 para el grupo >12 h, y el grupo ≤6 h mostró puntuaciones notablemente más altas (*p* < 0,001). Las puntuaciones en la GOSE a los 6 meses de la cirugía fueron de 4,05 ± 1,73 para el grupo ≤6 h, 3,05 ± 1,76 para el grupo 6–12 h y 3,19 ± 1,73 para el grupo >12 h, siendo las puntuaciones del grupo ≤6 h notablemente más altas (*p* = 0,001). La proporción de resultados favorables a los 6 meses de la operación fue del 47,3% para el grupo ≤6 h, del 24,6% para el grupo 6–12 h y del 18,8% para el grupo >12 h, siendo la proporción de resultados favorables más alta en el grupo ≤6 h (*p* = 0,001). La curva de supervivencia de Kaplan–Meier mostró que la tasa de supervivencia del grupo ≤6 h fue del 80,4%, significativamente superior al 57,9% del grupo 6–12 h y al 65,6% del grupo >12 h (F = 10,642, *p* = 0,005).

**Conclusión:**

La evacuación de un hematoma intracraneal mediante SMIS en las 6 horas siguientes al inicio de la hernia cerebral puede reducir de un modo eficaz el daño neurológico, mejorar significativamente las tasas de supervivencia y ofrecer un pronóstico favorable.

## 1. Introduction

The mortality rate of spontaneous intracerebral hemorrhage (sICH) is approximately 40%, with a disability rate as high as 75% [1]. Moreover, for patients with sICH combined with brain herniation, the in-hospital mortality rate was found to be as high as 60% [2]. A multicenter retrospective study on patients with sICH in the basal ganglia region found that the most effective surgical time frame for those with hematoma volumes between 30 and 50 mL was 6–12 h after the onset of bleeding. For individuals with hematoma volumes exceeding 50 mL, a time frame of ≤6 h was considered more beneficial [3]. Another study indicated that, for patients with supratentorial hematoma volumes >30 mL and a Glasgow coma scale (GCS) score of 5–12, the optimal surgical time window was considered to be between 7 and 24 h after the sICH [4]. Although previous studies have explored the time window for stereotactic minimally invasive surgery (SMIS) in sICH, there remains a research gap concerning patients with brain herniation [3]. The optimal surgical timing and procedure for patients with brain herniation remains unclear. To date, no study has reported the optimal time window for SMIS in patients with brain herniation. Our study aims to provide precise guidance for SMIS for treating brain herniation, which holds significant clinical application value and research importance.

## 2. Materials and Methods

### 2.1 Research Subjects

**Fig. 1.**
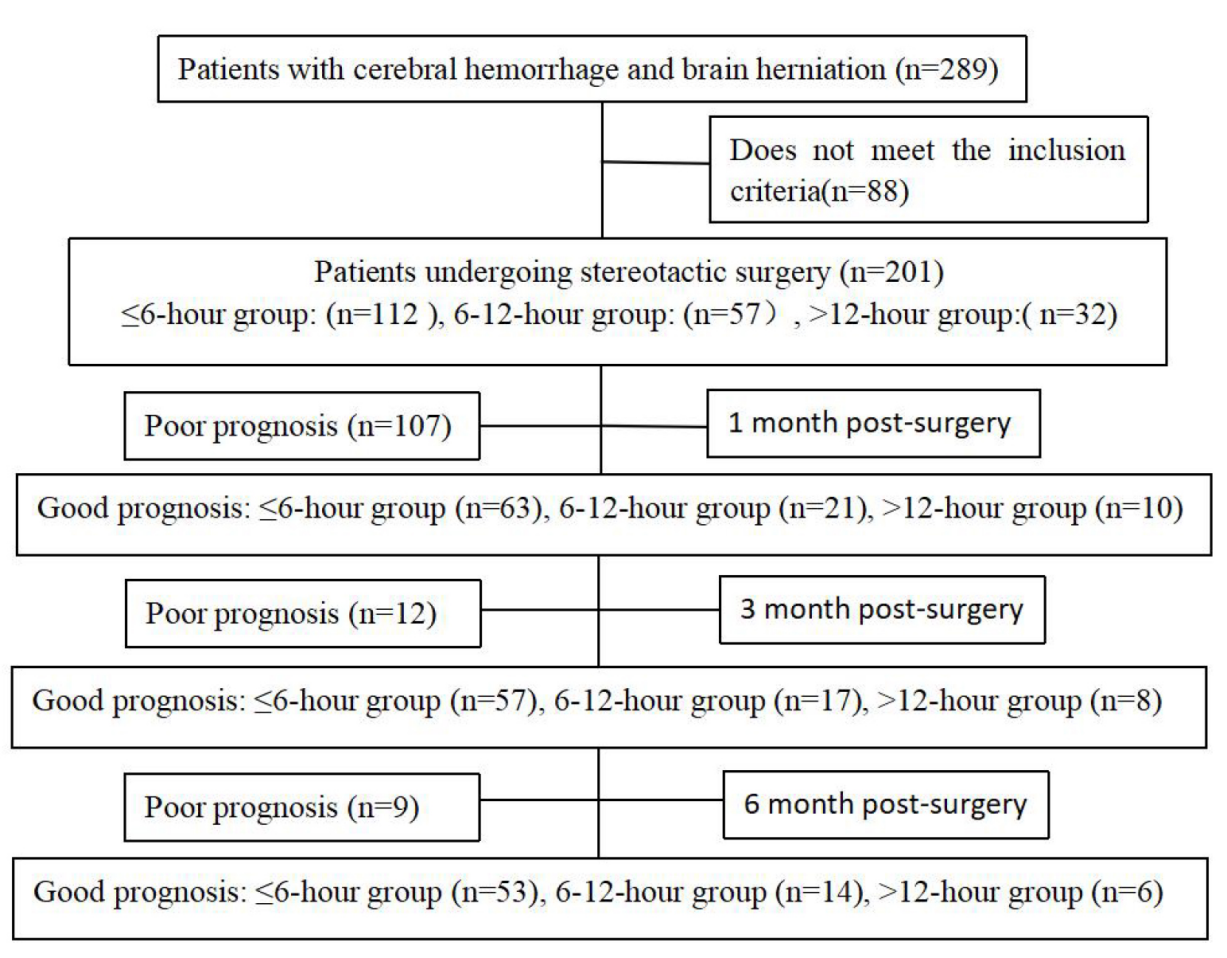
**Study flow of this trial**.

This retrospective study included 201 patients with sICH and brain herniation who were admitted to the Emergency Department of the Affiliated Hospital of Guizhou Medical University between January 2019 and October 2024. The patients were classified into three groups based on the time window from the occurrence of brain herniation to stereotactic surgery: ≤6-h, 6–12-h, and >12-h groups (Fig. 1). All enrolled patients were followed up for 6 months, and their prognoses and survival were assessed using the Glasgow Outcome Scale-Extended (GOSE), the Modified Rankin Scale (mRS), and mortality rates. Baseline clinical information was obtained from the hospital’s medical record system, and cranial computed tomography (CT) imaging data during hospitalization were obtained from the radiology department. Prognostic information was collected through telephone follow-up, and only patient samples with complete data were ultimately included in the study. Consent was obtained from all patients or their families, and the study was reviewed and approved by the Medical Ethics Committee of Guizhou Medical University Affiliated Hospital.

### 2.2 Definition of Brain Herniation

#### 2.2.1 Clinical Manifestations

The study participants presented with severe headache, frequent vomiting, and agitation, sometimes leading to impaired consciousness as the condition progressed. This was accompanied by dynamic changes in the pupils, characterized by initial constriction followed by progressive dilation of the pupil on the lesion side, unequal pupil sizes on both sides, and disappearance of the light reflex. These symptoms indicate a significant increase in intracranial pressure (ICP) and the formation of brain herniation.

#### 2.2.2 Intracranial Pressure (ICP)

When a hematoma exerts a mass effect on the brain, the cerebrospinal fluid volume and blood volume are reduced to maintain a normal ICP. However, as the volume of the lesion increases and exceeds the compensatory mechanisms of the ICP, the risk of brain herniation increases. A marked increase in ICP exceeding 20 mmHg within 6 h before the onset of clinical symptoms of brain herniation indicates that the intracranial system can no longer compensate for the increased volume, suggesting the formation of brain herniation. In the absence of ICP monitoring, an elevated ICP can be assessed by observing the following typical clinical manifestations: Persistent or paroxysmal severe headache, often worsening in the early morning, which may be accompanied by nausea and vomiting; temporary headache, which may occur following vomiting; and congestion and swelling of the optic disc, leading to blurred vision, visual field defects, or, in severe cases, blindness.

#### 2.2.3 Radiological Aspects

**Fig. 2.**
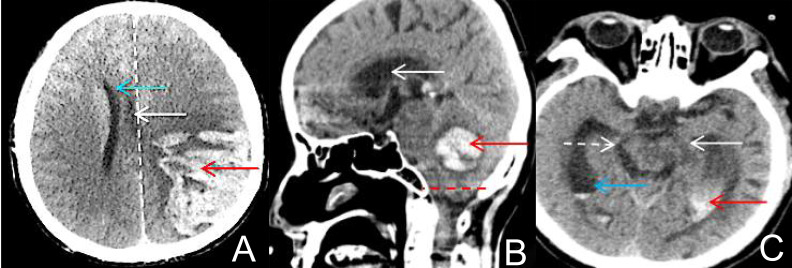
**Types of cerebral herniation**. (A) Subfalcine herniation: The left-sided hematoma (red arrow) compresses the brain tissue, pushing it toward the right, causing a deviation of the relative midline (dashed line) to the right. The third ventricle (white arrow) and lateral ventricle (blue arrow) are compressed by the brain tissue. (B) Tonsillar herniation: The sagittal view shows the hematoma (red arrow) compressing the brain tissue, which is pushed downward into the foramen magnum (red dashed line). Tonsillar herniation can also cause obstructive hydrocephalus in the fourth ventricle (white arrow). (C) Hippocampal gyrus herniation: The hematoma (red arrow) compresses the brain tissue and shifts it toward the cerebellar tentorial notch. The right basal cistern is widened (white dashed arrow), while the left basal cistern disappears, and the midbrain is displaced and rotated to the left (white arrow). A small amount of blood is present in the lateral ventricle, with expansion of the temporal horn of the lateral ventricle (blue arrow).

On computed tomography (CT) imaging (Fig. 2), midbrain displacement, compression, or even torsional deformation can be observed, accompanied by features such as unilateral or bilateral expansion of the temporal horn of the lateral ventricles, partial or complete obliteration of the surrounding cisterns, and the presence or absence of obstructive hydrocephalus [5]. Subfalcine herniation is the most common type of brain herniation. On CT imaging, a midline shift of brain tissue is observed, with the cingulate gyrus displaced under the falx cerebri, resulting in the formation of subfalcine herniation. The CT imaging characteristics of uncal herniation include the downward displacement of the hematoma along the tentorial notch, widening of the ipsilateral cistern, compression of the contralateral cistern, and contralateral dilation of the temporal horn of the lateral ventricle. Cerebellar tonsillar herniation is characterized on magnetic resonance imaging by a downward displacement of the cerebellar tonsils by more than 5 mm relative to the McRae line, along with evidence of obliteration of the cisterna magna, anterior displacement of the medulla, and associated hydrocephalus [5].

### 2.3 Inclusion and Exclusion Criteria

#### 2.3.1 Inclusion Criteria

The inclusion criteria were as follows: sICH primarily affects individuals with lobar hemorrhage, basal ganglia putaminal hemorrhage, or thalamic hemorrhage; CT imaging clearly indicates subfalcine herniation or uncal herniation; cranial CT showing a midline shift of ≥5 mm is indicative of increased intracranial pressure; and patients exhibiting unequal or irregularly shaped pupils, fixed gaze, sluggish or absent pupillary light reflex, and even Cheyne-Stokes respiration may also be experiencing severe neurological deficits; hematoma volume ≥30 mL.

#### 2.3.2 Exclusion Criteria

The exclusion criteria were as follows: Patients diagnosed with foramen magnum herniation confirmed by CT may present with brainstem or sICH with brain herniation, which can be caused by sICH resulting from the rupture of arteriovenous malformations; secondary sICH resulting from trauma, tumors, or cerebral infarction; and patients with incomplete or missing baseline data or follow-up information.

### 2.4 Definition of Postoperative Rebleeding

Postoperative rebleeding in patients can be determined based on the following criteria: Rebleeding typically occurs within 24 to 72 h after surgery; a hematoma volume increase of more than 33% or an absolute increase of more than 6 mL [6]; new bleeding foci, including bleeding that occurs outside the original surgical site, with CT imaging showing high-density areas; and rebleeding is usually accompanied by a worsening of clinical symptoms. The patient may experience exacerbation of preexisting neurological deficits, such as pupil dilation after returning to normal, worsening limb weakness or sensory disturbances, deepening impairment of consciousness from alertness to drowsiness, coma, aggravated headache, nausea, vomiting, or other symptoms of increased ICP.

### 2.5 Surgical Indications and Methods

**Fig. 3.**
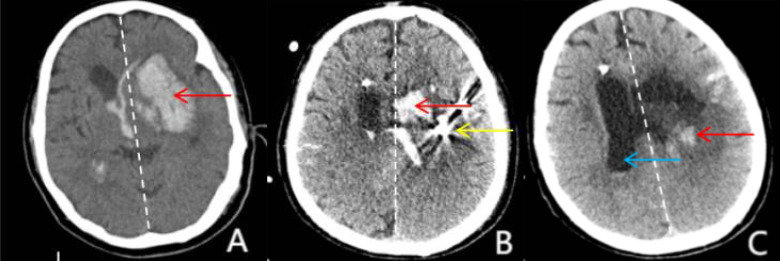
**Hematoma changes**. (A) CT on admission shows Compression by a left-sided hematoma (red arrow) causes brain tissue to shift to the right, with the midline (dotted line) displaced to the right (red arrow), indicating the formation of a subfalcine herniation. (B) Postoperative CT after SMIS shows a relative improvement in the midline (dotted line) compared to that in (A), with a significant reduction in hematoma residuals (red arrow). The stereotactic puncture needle (yellow arrow), placed during surgery, is visible and used for drainage or decompression. (C) Postoperative CT after removal of the puncture needle: The midline (dotted line) appears to have returned to near-normal alignment, indicating significant relief of brain herniation symptoms. The hematoma (red arrow) was nearly completely absorbed, and the right lateral ventricle (blue arrow) reappeared, suggesting that the compression of the lateral ventricle was resolved. CT, computed tomography; SMIS, stereotactic minimally invasive surgery.

The 2022 Guidelines for the Management of Spontaneous sICH from the American Stroke Association recommend SMIS or SMIS combined with thrombolysis for patients with supratentorial hemorrhage exceeding 20 mL in volume, a GCS score of 5–12, and progressive neurological decline [7]. Surgical intervention may be an option for comatose patients with supratentorial hemorrhage and large hematomas causing a significant midline shift or uncontrollable ICP to reduce mortality [7]. Previous study has indicated that surgical treatment can be considered for patients who meet the following criteria: a hematoma volume exceeding 30 mL, a midline shift greater than 0.5 cm, and significant compression of the lateral ventricle [8]. All patients in this study underwent CT-guided emergency stereotactic minimally invasive puncture and drainage SMIS, followed by postoperative injection of urokinase for hematoma drainage (Fig. 3). These procedures were crucial for ensuring successful patient outcomes.

### 2.6 Data Collection, Prognostic Evaluation, and Statistical Methods

#### 2.6.1 Data Collection and Prognostic Evaluation

Relevant data were collected from eligible patients, including sex, age, imaging findings, blood pressure, presence or absence of hypertension, presence or absence of diabetes, smoking history, alcohol consumption history, hematoma volume on admission, GCS score, National Institutes of Health Stroke Scale (NIHSS), mRS score, GOSE score, time from brain herniation to surgery, average ICP at 6 h post-surgery, white blood cell count, absolute eosinophil count, absolute basophil count, creatinine levels, and other related information. Therefore, the traditional Glasgow Outcome Scale (GOS) or mRS has limitations in evaluating the 180-day prognosis of patients. In contrast, the GOSE score offers significant advantages, particularly in groups with potentially poor outcomes, where it demonstrates higher calibration and predictive accuracy. A GOSE score of ≥4, which includes moderate disability, is defined as a favorable prognosis and provides a more scientific basis for evaluating a patient’s 180-day prognosis. The GOSE score has high sensitivity and precision, allowing for the graded assessment of functional outcomes, refining the functional status range from severe disability to full recovery. The method serves as a reliable tool for quantitative analysis of disease prognosis. Through telephone follow-up, the patients’ prognosis was assessed using the GOSE score, which evaluates the degree of disability and the 180-day clinical outcome. The GOSE score is divided into eight levels based on functional recovery, covering outcomes ranging from death to full recovery [9]. The prognostic evaluation based on the GOSE score was derived from a formula incorporating the patient’s GCS score upon admission, age, and hematoma volume. Patients were categorized into two groups, namely, the potentially favorable and potentially poor prognostic groups, using the following formula: 10 × GCS – age – 0.64 × hematoma volume [10]. The predetermined cutoff value was 27.67. Patients with a score 27.67 were classified into the potentially poor prognosis group, whereas those with a score >27.67 were classified into the potentially favorable prognosis group. The GOSE score ranged from 1 to 8. In the potentially favorable prognosis group, a GOSE score of ≥5 was considered favorable, whereas a score of ≤4 was considered poor prognosis. In the potentially poor prognosis group, a GOSE score of ≤4 was considered poor, whereas a score of ≥3 was considered favorable. The secondary outcomes of the patients’ prognoses were assessed using the 6-month survival rate and the mRS score to evaluate the degree of disability and level of dependence in daily living. The mRS score spans 0 to 6, where 0 represents no symptoms and 6 indicates death. An mRS score of 0–3 was defined as favorable prognosis, indicating a high degree of functional independence, whereas an mRS score of 4–6 was defined as poor prognosis, suggesting a higher level of dependency in daily life or death. The mRS score has universality and universality, and is currently the most widely used functional outcome indicator in the field of cerebral hemorrhage. The mRS score is concise and efficient, and can be completed through telephone follow-up, which can significantly reduce the dropout rate and is particularly suitable for this study. GOSE score, especially for patients over 60 years old with hematoma volume greater than 30 mL, has better long-term functional recovery. The GOSE score compensates for the shortcomings of mRS in evaluating work, social, and independent living abilities, and more accurately assesses the long-term quality of life of patients.

#### 2.6.2 Statistical Methods

Statistical analysis was conducted using SPSS version 26.0 (IBM SPSS statistics, Chicago, IL, USA). The normality of continuous variables was tested. Normally distributed measurement data are presented as the mean ± standard deviation ($\bar{\chi }$ ± s). One-way ANOVA (Analysis of Variance) was used for analysis, followed by post hoc pairwise comparisons: the LSD (Least Significant Difference) test was applied when variances were equal, and Tukey’s test when variances were unequal. Measurement data and ordinal data that did not follow a normal distribution were reported as the median (IQR) and analyzed using nonparametric tests (Mann–Whitney U test). Categorical data are presented as frequencies and proportions (%), and comparisons between groups were conducted using the chi-square (χ^2^) test or Fisher’s exact test. Kaplan–Meier survival analysis was used to evaluate survival over a 6-month period. A significance level of *p* < 0.05 was used to determine statistical significance.

## 3. Results

### 3.1 Baseline Data Analysis of Patients With sICH and Brain Herniation

We divided 201 patients with sICH and brain herniation into groups based on the time of SMIS: 112 cases in the ≤6-h group, 57 cases in the 6–12-h group, and 32 cases in the >12-h group. The three groups of patients showed no significant differences in sex, age, systolic blood pressure upon admission, diastolic blood pressure upon admission, smoking history, drinking history, history of diabetes, complications during hospitalization, vegetative state at discharge, GCS score upon admission, NIHSS score upon admission, hematoma volume upon admission, and other baseline data (Table 1). At admission, the GCS score at admission was 7.9 ± 3.8 points in the 6-h group, 7.7 ± 3.3 points in the 6–12-h group, and 9.4 ± 3.6 points in the >12-h group (Table 1, *p* = 0.082); the NIHSS score was 24.5 ± 8.9 in the 6-h group, 23.6 ± 8.4 in the 6–12-h group, and 20.0 ± 9.5 in the >12-h group (Table 1, *p* = 0.063); the age was 60.1 ± 12.4 years in the 6-h group, 61.2 ± 10.6 years in the 6–12-h group, and 59.6 ± 11.0 years in the >12-h group (Table 1, *p* = 0.76); and the hematoma volume was 70.0 ± 20.3 mL in the 6-h group, 68.1 ± 20.1 mL in the 6–12-h group, and 66.7 ± 24.2 mL in the >12-h group (Table 1, *p* = 0.695). The incidence of ventricle rupture was 85.7% in the ≤6-h group, 86% in the 6–12-h group, and 56.3% in the >12-h group. The incidence of ventricle rupture in the ≤6-h group and 6–12-h group was significantly higher than that in the >12-h group (Table 1, *p* < 0.05).

**Table 1.** **Baseline data analysis of patients with intracerebral hemorrhage and brain herniati**.

Variable		≤6 h (n = 112)	6–12 h (n = 57)	>12 h (n = 32)	F/χ^2^	*p*-value
Sex, male (n, %)		76 (67.9)	37 (64.9)	18 (56.3)	1.480	0.477
Age (years, $\bar{\chi }$ ± s)		60.1 ± 12.4	61.2 ± 10.6	59.6 ± 11.0	0.241	0.760
Systolic blood pressure at admission (mmHg, $\bar{\chi }$ ± s)		177.7 ± 33.5	169.4 ± 35.6	167.8 ± 28.8	1.745	0.177
Diastolic blood pressure at admission (mmHg, $\bar{\chi }$ ± s)		104.9 ± 21.0	99.8 ± 22.7	95.9 ± 19.8	2.668	0.072
Smoking history (n, %)		75 (67.0)	31 (54.4)	15 (46.9)	5.314	0.070
Drinking history (n, %)		71 (63.4)	43 (75.4)	18 (56.3)	3.930	0.140
History of diabetes (n, %)		7 (6.3)	7 (12.3)	2 (6.3)	2.027	0.363
History of stroke (n, %)		13 (11.6)	5 (8.8)	1 (3.1)	2.135	0.344
	History of hyperlipidemia (n, %)	1 (0.9)	3 (5.3)	0 (0.0)	4.472	0.107
	History of coronary heart disease (n, %)	6 (5.4)	1 (1.8)	0 (0.0)	2.832	0.243
	History of hypertension (n, %)	89 (79.5)	41 (71.9)	23 (71.9)	1.557	0.459
	Ventricular involvement (n, %)	96 (85.7)	49 (86.0)	18 (56.3)	15.324	<0.001
	Complications encountered during hospitalization (n, %)	73 (65.2)	35 (61.4)	19 (59.4)	3.778	0.707
	Average postoperative ICP in 6 h [mmHg, M (Q_1_∼Q_3_)]	12.4 [9.6, 15.4]	13.7 [9.8, 16.4]	13.7 [10.5, 17.0]	2.961	0.228
	Hematoma volume at admission (mL, $\bar{\chi }$ ± s)	70.0 ± 20.3	68.1 ± 20.1	66.7 ± 24.2	0.364	0.695
	Vegetative state at discharge (n, %)	13 (11.6)	14 (24.6)	4 (12.5)	5.009	0.082
	Secondary epilepsy at discharge (n, %)	1 (0.9)	1 (1.8)	1 (3.1)	0.881	0.644
	Absolute neutrophil-to-lymphocyte ratio [M (Q_1_∼Q_3_)]	10.9 [4.1, 16.3]	9.9 [5.3, 16.0]	7.4 [4.9, 12.8]	0.326	0.850
	Absolute neutrophil count [×10^9^/L, M (Q_1_∼Q_3_)]	9.7 [6.9, 13.4]	8.4 [6.3, 11.7]	8.1 [6.3, 11.4]	1.967	0.374
	Absolute lymphocyte count [×10^9^/L, M (Q_1_∼Q_3_)]	1.0 [0.7, 1.7]	0.9 [0.7, 1.6]	1.0 [0.8, 1.4]	0.400	0.819
	Absolute eosinophil count [×10^9^/L, M (Q_1_∼Q_3_)]	0.0 [0.0, 0.1]	0.0 [0.0, 0.1]	0.0 [0.0, 0.1]	0.276	0.871
	Absolute basophil count [×10^9^/L, M (Q_1_∼Q_3_)]	0.0 [0.0, 0.1]	0.0 [0.0, 0.0]	0.0 [0.0, 0.0]	4.078	0.130
	Platelet count [×10^9^/L, M (Q_1_∼Q_3_)]	184.0 [149.0, 241.0]	184.0 [149.0, 234.0]	203.0 [157.0, 229.0]	0.107	0.948
	White blood cell count [×10^9^/L, M (Q_1_∼Q_3_)]	11.9 [8.8, 15.1]	10.2 [8.4, 13.1]	9.8 [8.4, 12.7]	5.773	0.056
Creatinine [µmol/L, M (Q_1_∼Q_3_)]		70.5 [56.7, 83.0]	64.4 [47.2, 94.4]	60.5 [46.9, 83.0]	3.088	0.214
Alanine aminotransferase [U/L, M (Q_1_∼Q_3_)]		19.3 [13.2, 27.6]	18.7 [13.6, 24.5]	16.7 [13.0, 21.8]	1.494	0.474
	Aspartate aminotransferase [U/L, M (Q_1_∼Q_3_)]	24.7 [20.3, 33.0]	25.3 [19.5, 31.4]	22.3 [18.1, 29.0]	2.092	0.351
	Total protein content [g/L, M (Q_1_∼Q_3_)]	70.4 [65.9, 75.0]	70.5 [64.7, 76.0]	68.0 [60.6, 72.1]	3.663	0.160
	Potassium [mmol/L, M (Q_1_∼Q_3_)]	3.6 [3.3, 3.9]	3.5 [3.4, 3.9]	3.8 [3.5, 3.9]	1.999	0.368
	Sodium [mmol/L, M (Q_1_∼Q_3_)]	141.0 [138.2, 142.7]	141.6 [139.0, 144.6]	142.3 [140.1, 144.1]	2.681	0.262
	Calcium [mmol/L, M (Q_1_∼Q_3_)]	2.2 [2.1, 2.3]	2.2 [2.0, 2.3]	2.2 [2.1, 2.3]	1.302	0.521
	Activated partial thromboplastin time [s, M (Q_1_∼Q_3_)]	31.4 [28.1, 35.5]	31.0 [28.6, 35.2]	33.0 [28.9, 37.8]	1.880	0.391
	Hematoma clearance rate (%, $\bar{\chi }$ ± s)	52.23 ± 38.19	48.29 ± 27.51	49.40 ± 30.54	0.195	0.907
	Postoperative rebleeding (n, %)	15.0 (13.39)	9.0 (15.79)	3.0 (9.38)	1.524	0.822
	Length of hospital stay [days, M (Q_1_∼Q_3_)]	15.0 [10.0, 25.0]	14.0 [8.0, 22.0]	11.0 [9.0, 14.0]	4.576	0.101
	GCS score upon admission (points, $\bar{\chi }$ ± s)	7.9 ± 3.8	7.7 ± 3.3	9.4 ± 3.6	4.998	0.082
NIHSS score upon admission (points, $\bar{\chi }$ ± s)		24.5 ± 8.9	23.6 ± 8.4	20.0 ± 9.5	5.529	0.063

Note: Continuous variables are expressed as the mean ± SD or as the median (interquartile range). Categorical variables are expressed as frequency (percentage). ICP, intracranial pressure; M, median; GCS, Glasgow coma scale; NIHSS, National Institutes of Health Stroke Scale.

### 3.2 Analysis of mRS Scores

The mRS scores at 1, 3, and 6 months postoperatively were 3.72 ± 1.03, 3.66 ± 1.14, and 3.71 ± 1.30, respectively, in the ≤6-h group; 4.37 ± 0.99, 4.42 ± 1.02, and 4.61 ± 1.25, respectively, in the 6–12-h group; and 4.09 ± 1.09, 3.97 ± 1.31, and 4.18 ± 1.35, respectively, in the >12-h group. The mRS scores of the ≤6-h group were notably lower than those of the 6–12-h group and the >12-h group (Table 2, *p* < 0.05). Surgery within 6 h significantly improved neurological functional prognosis, further highlighting the importance of optimizing the surgical time window.

**Table 2.** **Analysis of mRS scores**.

Variable	≤6 h (n = 112)	6–12 h (n = 57)	>12 h (n = 32)	F	*p*-value
mRS score 1 month after surgery (points, $\bar{\chi }$ ± s)	3.72 ± 1.03*	4.37 ± 0.99	4.09 ± 1.09	7.695	0.001
mRS score 3 months after surgery (points, $\bar{\chi }$ ± s)	3.66 ± 1.14*	4.42 ± 1.02	3.97 ± 1.31	8.541	<0.001
mRS score at 6 months after surgery (points, $\bar{\chi }$ ± s)	3.71 ± 1.30*	4.61 ± 1.25	4.18 ± 1.35	9.315	<0.001

Note: *compared with 6–12-h group, *p* < 0.05. mRS, Modified Rankin Scale.

### 3.3 Analysis of GOSE Scores and Favorable Outcomes

The GOSE scores at 1, 3, and 6 months postoperatively were as follows: the scores of the ≤6-h group were 4.19 ± 1.37, 4.17 ± 1.48, and 4.05 ± 1.73, respectively; those of the 6–12-h group were 3.54 ± 1.43, 3.39 ± 1.58, and 3.05 ± 1.76, respectively; and those of the >12-h group were 3.84 ± 1.39, 3.81 ± 1.45, and 3.19 ± 1.73, respectively. The GOSE scores of the ≤6-h group were significantly higher than those of the 6–12-h group and the >12-h group (Table 3, *p* < 0.05). The proportions of patients with good prognosis at 1, 3, and 6 months after surgery were 56.3%, 50.9%, and 47.3% in the ≤6-h group; 36.8%, 29.8%, and 24.6% in the 6–12-h group; and 32.3%, 25.0%, and 18.8% in the >12-h group. The proportion of patients with good prognosis was higher in the ≤6-h surgical group than in the 6–12-h group and the >12-h group (Table 3, *p* < 0.05). This indicates that surgical intervention within ≤6 h can significantly improve the functional 6-month prognosis of patients.

**Table 3.** **Analysis of GOSE scores and favorable outcomes**.

Variable		≤6 h (n = 112)	6–12 h (n = 57)	>12 h (n = 32)	F/χ^2^	*p*-value
GOSE score 1 month after surgery (points, $\bar{\chi }$ ± s)		4.19 ± 1.37*	3.54 ± 1.43	3.84 ± 1.39	4.656	0.011
GOSE score 3 months after surgery (points, $\bar{\chi }$ ± s)		4.17 ± 1.48*	3.39 ± 1.58	3.81 ± 1.45	5.499	0.005
GOSE score 6 months after surgery (points, $\bar{\chi }$ ± s)		4.05 ± 1.73*	3.05 ± 1.76	3.19 ± 1.73^★^	7.543	0.001
	Good prognosis at 1 month (n, %)	63 (56.3)*	21 (36.8)	10 (31.3)^★^	9.396	0.009
	Good prognosis at 3 months (n, %)	57 (50.9)*	17 (29.8)	8 (25.0)^★^	10.874	0.004
	Good prognosis at 6 months (n, %)	53 (47.3)*	14 (24.6)	6 (18.8)^★^	13.540	0.001
6-month survival rate (n, %)		90 (80.4)*	33 (57.9)^#^	21 (65.6)^★^	10.060	0.007

Note: *compared with 6–12-h group, *p* < 0.05. ^#^compared with >12-h group, *p* < 0.05. ^★^compared with 6-h group, *p* < 0.05. GOSE, Glasgow Outcome Scale Extended.

### 3.4 Kaplan–Meier Survival Analysis

**Fig. 4.**
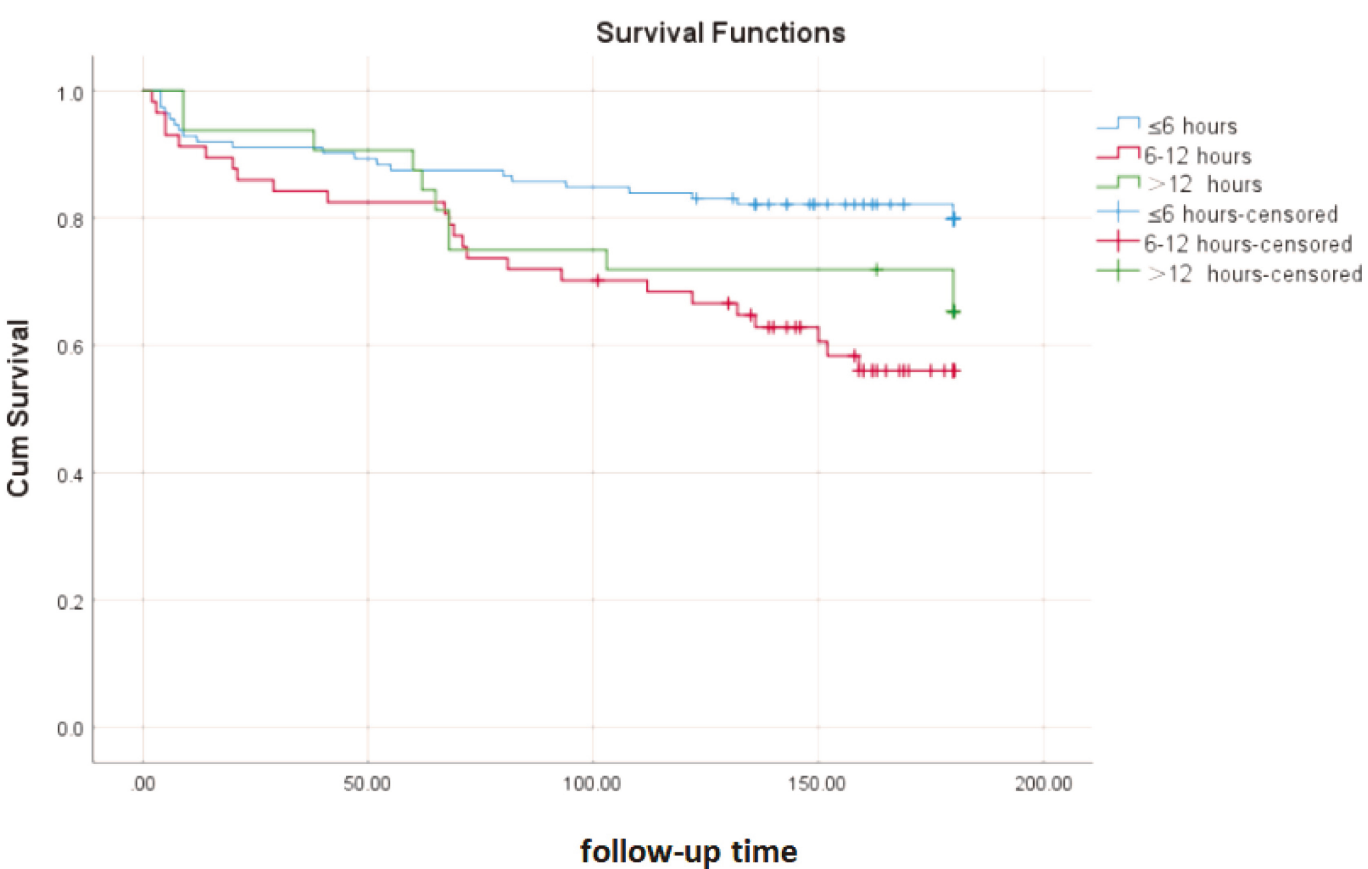
**Kaplan–Meier survival curve for the time window of minimally invasive stereotactic treatment**.

In the group with a time frame of ≤6 h, 90 cumulative patients survived, resulting in a survival rate of 80.4%. In contrast, the group with a time frame of 6–12 h had 33 cumulative surviving patients, resulting in a survival rate of 57.9%. Lastly, in the group with a time frame exceeding 12 h, 21 cumulative surviving patients were recorded, with a survival rate of 65.6% (Table 3, *p* = 0.007). The Kaplan–Meier survival curve analysis indicated that the survival rate of patients who had surgery within ≤6 h was significantly higher than that of the 6–12-h group and the >12-h group, with a significant difference (F = 10.642, *p* = 0.005, Fig. 4). Additional analysis indicated that as the time from brain herniation to surgical intervention increased, the cumulative survival of patients gradually decreased. This indicates that early intervention plays a crucial role in improving patient survival rates.

## 4. Discussion

SMIS combined with thrombolytic therapy is a commonly used method for hematoma evacuation, offering advantages such as simplicity, minimal trauma, shorter operative time, and significant efficacy, while its CT-guided precise localization and removal of hematomas help alleviate mass effects and reduce postoperative complications, providing a safe and efficient option for clinical treatment [11, 12]. However, the timing of surgery still requires a comprehensive assessment based on the patient’s condition and other relevant factors. Current research and clinical practice typically recommend surgical intervention within 24 h after onset or during the ultra-early stage of onset, which is within 6 h. Several study has indicated that the rate of rebleeding significantly increases in patients with sICH who undergo surgery within 3 or 5 h after onset [13]. Our study indicates that the proportion of favorable outcomes and survival rates in the ≤6-h group were higher than those in the other two groups. The incidence of postoperative rebleeding was 13.39% in the ≤6-h group, 15.79% in the 6–12-h group, and 9.38% in the >12-h group, with no significant differences among the three groups. According to a multicenter retrospective study report, for patients with hematoma volumes exceeding 50 mL, a time window of ≤6 h may offer benefits, which is consistent with our findings [3]. The hematoma volume was 70.0 ± 20.3 mL in the ≤6-h group, 68.1 ± 20.1 mL in the 6–12-h group, and 66.7 ± 24.2 mL in the >12-h group, with no significant differences among the three groups. However, despite having larger hematoma volumes, the ≤6-h group showed better patient outcomes than both the 6–12-h and >12-h groups. A 2021 prospective study found that performing surgery within 6 h of sICH onset was beneficial for treating severe hypertensive sICH, and this approach effectively improved neurological function, daily living ability, motor function, quality of life, and prognosis, which is consistent with our own findings [14]. The hematoma clearance rates were 52.23 ± 38.19% in the ≤6-h group, 48.29 ± 27.51% in the 6–12-h group, and 49.40 ± 30.54% in the >12-h group. Notably, the ≤6-h group demonstrated superior outcomes in hematoma clearance compared to both the 6–12-h and >12-h groups.

The results of this study indicate that hypertensive patients are more prone to ICH. However, study suggests that non-hypertensive mechanisms also play an important role in the pathogenesis of sICH. Cerebral amyloid angiopathy (CAA), the most common pathological change in cerebral small vessel disease aside from arteriosclerosis, has been found to play a significant role in non-hypertensive ICH and cognitive decline [15]. The diagnostic rate of CAA has greatly improved in recent years due to advancements in imaging technology and the identification of new imaging markers [15]. Lobar hematoma is the most serious complication of CAA, with a total mortality rate of 10%–40% in patients with CAA-related ICH and an annual recurrence risk of approximately 10% [16].

A previous study suggested that thalamic hemorrhage accounted for 1.4% of all stroke cases and 13% of ICH cases, while hypertension (53.2%), vascular malformations (6.4%), hematological conditions (4.3%), and anticoagulation 2.1% were the main causes of thalamic hemorrhage [17]. Altered consciousness, intraventricular extension of the hematoma, and advanced age were identified as key determinants of a poor early outcome [17]. These findings are especially relevant for patients presenting with impaired consciousness, intraventricular hemorrhage, and advanced age, and they help improve the prognosis of patients, providing important evidence for optimizing the surgical time window of SMIS in patients with brain herniation. A detailed explanation of the patients’ causes of death is provided in the Discussion section. During hospitalization and follow-up, a total of 57 patients died from various causes. The neurological causes of death were as follows: 15 patients died from brain herniation, which caused a sharp increase in ICP, leading to the compression of brain tissue and nerves, resulting in brain dysfunction; among them, 8 patients underwent a follow-up head CT scan, which revealed significant cerebral edema. Secondary massive cerebral edema further exacerbated ICP, affected cerebral blood flow and function, and ultimately led to death. The non-neurological causes of death were as follows: 18 patients died from lung infections and respiratory failure; 8 patients died due to postoperative bleeding; 3 patients died from secondary epilepsy; 4 patients died from acute heart failure and underlying heart disease; and 1 patient died due to combined liver failure, kidney failure, and coagulation dysfunction.

Although this study provides new insights into the optimal timing for SMIS for treating ICH with brain herniation, it also has several limitations. First, due to its retrospective design, there is potential for selection bias and information bias. Second, the relatively small sample size may limit the generalizability and external validity of the results. Future studies should consider using prospective randomized controlled trials to reduce bias and improve the reliability of the results. Expanding the sample size and increasing the number of participating centers could further enhance the study’s generalizability. Additionally, future research should explore other factors that affect surgical timing and postoperative outcomes.

Although there is existing literature on the timing of surgical treatment for cerebral hemorrhage, research specifically on the use and timing of minimally invasive procedures for brain herniation is limited. In this research, we aimed to fill this gap by investigating the optimal timing of SMIS for treating brain herniation. The novelty of this study can be reflected in several aspects. Firstly, we systematically investigated the timing of SMIS in treating brain herniation, thus filling a gap in the current literature. Second, unlike previous studies, this research focused solely on SMIS as a treatment option, offering a fresh perspective compared to traditional craniotomy and endoscopic surgery [18]. Lastly, by conducting long-term follow-ups, we evaluated the impact of surgical timing on long-term outcomes, providing valuable insights into the lasting effects of SMIS for brain herniation. The exploration of the time window for SMIS in the treatment of brain herniation in this study not only provides new guidance for clinical practice but also provides a direction for future research, with significant clinical application value and research significance. Future efforts should further focus on predicting hematoma expansion preoperatively and optimizing the surgical time window to enhance the safety and efficacy of the procedure and promote the clinical application of more optimal treatment strategies.

## 5. Conclusion

In summary, undergoing SMIS within 6 h can significantly improve long-term outcomes and increase survival rates among patients with acute sICH complicated by brain herniation. The intervention of SMIS is not only beneficial for postoperative functional recovery, but also has significant importance for long-term survival. This study provides important evidence for optimizing the treatment time window and surgical approach for patients with acute supratentorial cerebral hemorrhage and brain herniation.

## Data Availability

The datasets collected and analyzed during this study are available from the corresponding author [LKW] upon reasonable request.

## Abbreviations

sICH, supratentorial intracerebral hemorrhage; CT, computed tomography; SMIS, stereotactic minimally invasive surgery; ICP, intracranial pressure; GCS, Glasgow Coma Scale; GOS, Glasgow Outcome Scale; GOSE, Glasgow Outcome Scale Extended; NIHSS, National Institute of Health Stroke Scale; mRS, Modified Rankin Scale; CAA, Cerebral amyloid angiopathy.

## Supplementary Material

Supplementary material associated with this article can be found, in the online version, at https://doi.org/10.31083/RN38627.
